# Genome-Wide Identification, Evolution, and Expression Analysis of LBD Transcription Factor Family in Bread Wheat (*Triticum aestivum* L.)

**DOI:** 10.3389/fpls.2021.721253

**Published:** 2021-09-03

**Authors:** Zhenyu Wang, Ruoyu Zhang, Yue Cheng, Pengzheng Lei, Weining Song, Weijun Zheng, Xiaojun Nie

**Affiliations:** ^1^State Key Laboratory of Crop Stress Biology in Arid Areas, College of Agronomy and Yangling Branch of China Wheat Improvement Center, Northwest A&F University, Yangling, China; ^2^Australia-China Joint Research Centre for Abiotic and Biotic Stress Management in Agriculture, Horticulture and Forestry, Yangling, China

**Keywords:** abiotic stress, expression profile, genetic variation, LBD gene family, wheat

## Abstract

The lateral organ boundaries domain (LBD) genes, as the plant-specific transcription factor family, play a crucial role in controlling plant architecture and stress tolerance. Although it has been thoroughly characterized in many species, the LBD family was not well studied in wheat. Here, the wheat LBD family was systematically investigated through an *in silico* genome-wide search method. A total of 90 wheat LBD genes (TaLBDs) were identified, which were classified into class I containing seven subfamilies, and class II containing two subfamilies. Exon–intron structure, conserved protein motif, and cis-regulatory elements analysis showed that the members in the same subfamily shared similar gene structure organizations, supporting the classification. Furthermore, the expression patterns of these TaLBDs in different types of tissues and under diverse stresses were identified through public RNA-seq data analysis, and the regulation networks of TaLBDs involved were predicted. Finally, the expression levels of 12 TaLBDs were validated by quantitative PCR (qPCR) analysis and the homoeologous genes showed differential expression. Additionally, the genetic diversity of TaLBDs in the landrace population showed slightly higher than that of the genetically improved germplasm population while obvious asymmetry at the subgenome level. This study not only provided the potential targets for further functional analysis but also contributed to better understand the roles of LBD genes in regulating development and stress tolerance in wheat and beyond.

## Introduction

The lateral organ boundaries domain (LBD), also called the AS2/LOB gene family, is one class of plant-specific transcription factors, which plays an important role in regulating lateral organ development, morphogenesis, and metabolism in plants (Majer and Hochholdinger, 2011). LBD genes are expressed in a band of cells at the adaxial base of all lateral organs formed from the shoot apical meristem and at the base of lateral roots. LBD transcription factors are typically defined by an N-terminal LBD domain, which generally comprises a C-domain containing four highly conserved cysteine (C) residues in a CX2CX6CX3C zinc finger-like motif. Besides, some LBD proteins also possessed the glycine residue and leucine zipper-like motif LX6LX3LX6L (Shuai et al., 2002; Matsumura et al., 2009). Generally, the LBD proteins can be divided into class I and class II subfamily according to their conserved domains. In detail, class I members usually contain four highly conserved cys residues in a CX2CX6CX3C motif, together with a leucine zipper-like motif (LX6LX3LX6L) that can form the coiled-coiled structure for protein interactions, while class II members have the conserved cys residues and leucine zipper-like domain that cannot form the coiled-coil structure (Landschaslz, 1998). The majority of LBD proteins belong to class I and widely participate in regulating plant development and signal transduction (Majer and Hochholdinger, 2011; Yu et al., 2020).

Up to now, extensive studies have been conducted to investigate the LBD gene family in many plants, such as 34 putative LBD genes found in barley, 44 in maize, and 35 in rice, respectively (Yang et al., 2006; Zhang et al., 2014; Guo et al., 2016). Meanwhile, the biological function of some LBD genes has been well studied. In Arabidopsis, AS1 and AS2 genes were found to be involved in the establishment of a prominent midvein and other veins and in the formation of the symmetric leaf lamina, which might be related to the repression of knotted 1 homeobox genes in leaves (Semiarti et al., 2001). *AtLBD16* and *AtLBD18* functioned in the initiation and emergence of lateral root formation *via* different pathways, and they could interact with downstream ARF7 and ARF19 (Lee et al., 2009). *AtASL9* was found to be exclusively regulated by the plant hormone cytokinin (Naito et al., 2007). In rice, *OsARL1*, encoded the protein with an LBD domain, was an auxin-responsive factor involved in the auxin-mediated cell dedifferentiation (Liu et al., 2005). *OsAS2* was required for shoot differentiation and leaf development (Ma et al., 2009). *OsLBD3-7* might act as the upstream regulatory gene of bulliform cell development to regulate leaf rolling (Li et al., 2016). In soybean, the expression of *GmLBD12* could be induced by drought, salt, cold, and hormones (Yang et al., 2017).

Wheat is one of the most important crops all over the world, occupying approximately 17% of global cultivated lands (Shewry, 2009). Wheat provides plenty of proteins, starches, and vitamins for humans, acting as an essential plant protein resource. Genetically, wheat is an allohexaploid, derived from three diploid donor species through two naturally interspecific hybridization events (Berkman et al., 2013; Thomas et al., 2014). As a result, wheat has a huge and complicated genome with three subgenomes (A, B, and D), making it an ideal model for polyploidization and homoeologous gene interaction studies in plants (Wicker et al., 2011). The completion of its reference sequence provides the opportunity to investigate the genomic organization and evolution dynamics of the wheat gene family at the genomic level (IWGSC, 2018). At present, the systematical investigation of the LBD gene family has not yet been performed in wheat. Here, we conducted an *in silico* genome-wide search of the LBD gene family in wheat. Then, the phylogenetic relationship, chromosome localization, gene structure, conserved protein domain, cis-elements, expression profiles, and regulatory network and genetic variations of the putative wheat LBD genes (TaLBDs) were systematically analyzed. These results provided global information on the composition, structure, and evolution of the TaLBD family, which will facilitate the further functional study of this important transcript factor family in wheat and beyond.

## Materials and Methods

### Databases Search and Sequences Analysis

The reference sequence (release-49) of the Chinese Spring genome and its annotated proteins were downloaded from the EnsemblPlants database (ftp://ftp.ensemblgenomes.org/pub/plants/release_49/fasta/triticum_aestivum/) (IWGSC, 2018). Then, two methods were adopted to identify putative TaLBDs. Firstly, the LBD proteins of rice and Arabidopsis were retrieved and downloaded from RGAP (http://rice.plantbiology.msu.edu/index.shtml) and TAIR (https://www.arabidopsis.org/) databases, respectively, and then used as the queries to perform a BLASTP search against the local protein database of wheat (IWGSC_v1.1) with an e-value of 1e-5 and identity of 50% as the threshold. Furthermore, the LBD domain (PF03195) obtained from the PFAM database (http://pfam.xfam.org/) was used as the query for the hidden Markov model (HMM) search using HMMER 3.0 program (http://hmmer.org/). The protein sequences identified by both aforementioned methods were integrated and parsed by manual editing to remove the redundancy. The remaining proteins were considered as the putative TaLBDs. The chromosome locations of these LBD genes were obtained by referring the gff annotation file and then were visualized using the Circos tool (v0.67) (Krzywinski et al., 2009). Finally, the putative TaLBDs were submitted to the NCBI-CDD server (http://www.ncbi.nlm.nih.gov/Structure/cdd/wrpsb.cgi) and the SMART database (http://smart.embl.de/) to confirm the existence of the LBD domain. The theoretical isoelectric point (PI) and molecular weight (MW) of the identified LBD proteins were calculated by the ExPASy server (http://www.expasy.org/). The subcellular localization prediction of each protein was predicted using the cello software (Yu et al., 2006).

### Phylogenetic, Gene Structure, and Conserved Motif Analysis

All of the identified TaLBD proteins together with LBD proteins of rice and Arabidopsis were used to perform a multiple sequence alignment using the DNAMAN tools (Woffelman, 2004) with the default parameters. An unrooted neighbor-joining tree with 1,000 bootstrap replications was constructed using MEGA 8.0 (Kumar et al., 2018) based on the full-length protein alignment. The exon–intron organizations and splicing phase of these predicted LBD genes were retrieved from the annotation file of the wheat genome and then graphically displayed by the Gene Structure Display Server (http://gsds.cbi.pku.edu.cn/). Conserved motifs or domains were predicted using the MEME tool (http://meme-suite.org/), with the following parameters: the maximum number of motifs set at 15 and the optimum width of each motif falls between 5 and 200 residues.

### Promoter Analysis and Identification of miRNAs Targets

The upstream 1.5 kilobases (kb) genomic DNA sequences of each predicted TaLBDs were extracted from the wheat genome and then submitted to the PlantCare database (http://bioinformatics.psb.ugent.be/webtools/plantcare/) to identify the putative cis-regulatory elements in the promoter regions. Furthermore, all the identified LBD transcripts were searched against the published wheat microRNAs (miRNAs) in the miRBase using the psRNATarget tool (Dai et al., 2018) to predict the TaLBDs targeted by miRNA.

### Gene Expression and Regulatory Network Analysis

To study the expression profiles of TaLBDs, a total of 49 public available RNA-seq samples from five tissues (root, stem, leaf, spike, and grain) and four stressed conditions (cold, salt, heat, and drought) were downloaded from WHEAT URGI (https://urgi.versailles.inra.fr/files/RNASeqWheat/) and NCBI Sequence Read Archive (SRA) database. The accession numbers and sample information were listed in Supplementary Table 1. The FPKM value (fragments per kilobase of transcript per million fragments mapped) was calculated for each LBD gene by Hisat2 and Stringtie software (Kim et al., 2015) and the spatial-temporal expression patterns of them were obtained. The heatmap was drawn based on the log 10-transformed (FPKM + 1) values through pheatmap package in R software.

To obtain the regulation relationship between TaLBDs and other wheat genes, the interaction networks, where these putative TaLBDs were involved in, were investigated based on the orthologous genes between wheat and Arabidopsis using the STRING tool (http://string-db.org/cgi) and the AraNet V2 tool (http://www.inetbio.org/aranet/). The predicted interaction network was displayed through the Cytoscape software (Otasek et al., 2019).

### Genetic Variations of TaLBDs

The genetic diversity of the LBD family in wheat landrace and genetically improved germplasm populations was investigated based on the resequencing data (Zhou et al., 2020). VCF file was downloaded from the Genome Variation Map (https://bigd.big.ac.cn/gvm) and the single-nucleotide polymorphisms (SNPs) in TaLBDs were extracted from the VCF file. Then, the nucleotide diversity (Pi value) in landrace and genetically improved germplasm populations was calculated using the vcftools (v 0.1.16).

### qRT-PCR Validation of TaLBDs Under Salt Stress

Quantitative PCR analysis was performed using the previously described method with some modifications (Lei et al., 2021). In brief, the seeds of cv. Chinese Spring were germinated in Petri dishes and grown in water at a growth chamber with the controlled condition (23°C ± 1°C, 16-h light/8-h dark cycle). The three-leaf seedlings were subjected to salt stress treatment. The healthy plants were incubated in 150 mM NaCl solution for 6, 12, and 24 h with the plants under normal conditions used as the control. Leaves of all these samples were collected with three biological replications. Total RNA was isolated by Plant RNA Kit reagent (Omega Bio-Tek, GA, USA) according to the instructions of the manufacturer. A total of 12 TaLBDs belonging to four homoeologous groups were selected to investigate their expression levels under salt stress conditions by QPCR analysis using the primers as listed in Supplementary Table 2. QPCR reactions were performed using the QuantStudioTM 7 Flex platform (Thermo Fisher Scientific, USA) with SYBR® Premix Ex Taq™ II reagent following the manufacturer's protocol (TaKaRa, Dalian, China). The thermal cycling condition of QPC analysis was as follow: 95°C for 30 s followed by 40 cycles of 95°C for 3 s, 60°C for 30 s.

## Results and Discussion

### Identification of the LBD Family in Wheat

**Figure 1:**
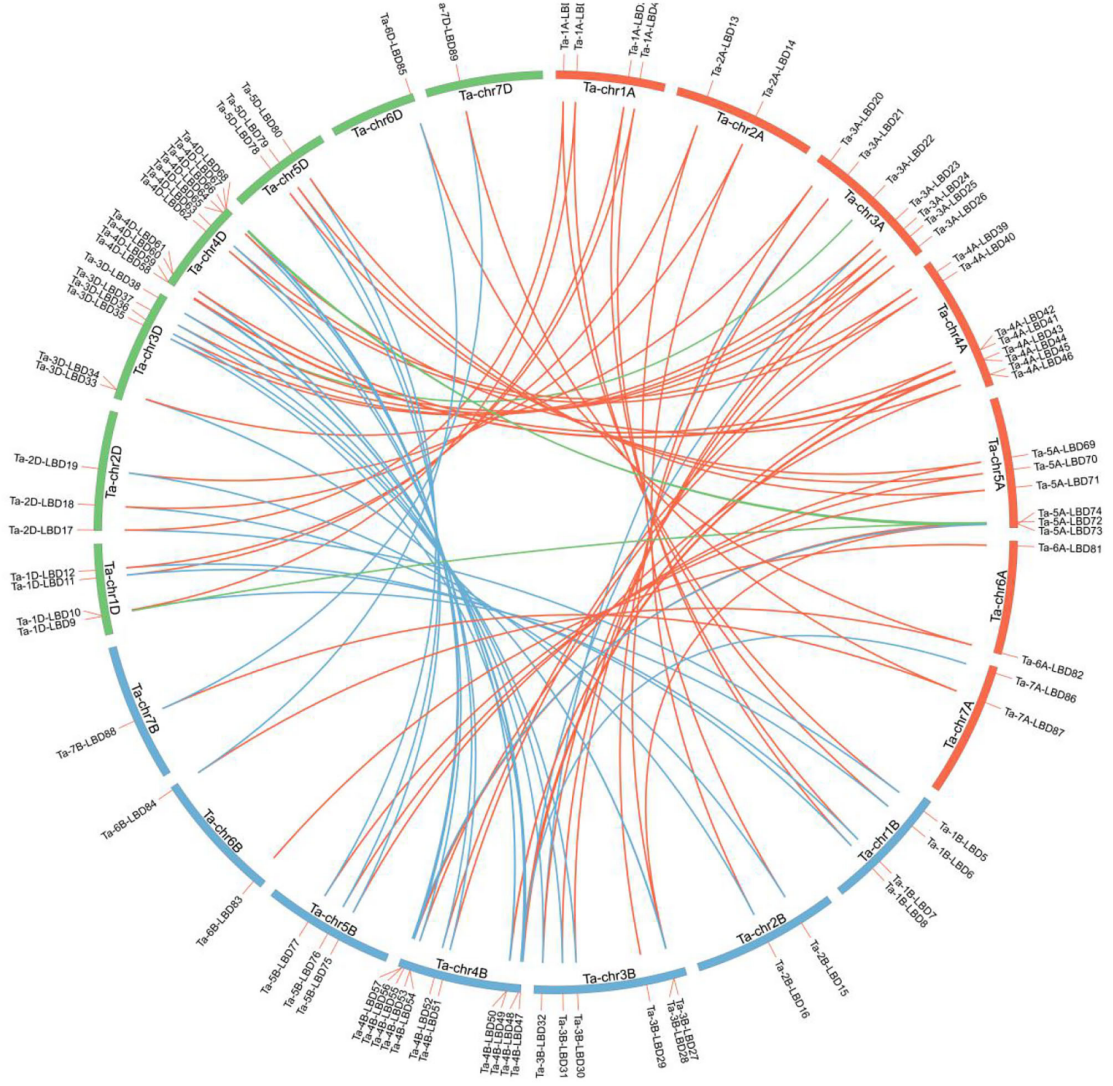
Distribution of the identified 90 TaLBDs across the seven chromosome groups and A (red), B (blue), and D (green) subgenomes of wheat. All wheat chromosomes are drawn to scale based on their actual physical lengths. TaLBDs, wheat lateral organ boundaries domain genes.

Using the aforementioned method, a total of 90 putative LBD proteins were identified in the wheat genome, which represented the most abundant LBD family among plants, indicating that the LBD gene family expanded significantly in wheat (Tables 1, 2). Chromosome location analysis showed that 89 TaLBDs were unevenly distributed on all of the 21 wheat chromosomes and only one (Ta-U-LBD90) was not mapped on any chromosome, of which 4B and 4D had the most abundant LBD genes with each containing 11, followed by 4A with 8, 3A with 7, and 3B, 3D, and 5A with 6, respectively, while 6B, 7B, and 7D only possessed one LBD gene (Figure 1). A total of 31, 29, and 29 LBD genes are non-randomly distributed in the wheat A, B, and D subgenomes, respectively, indicating that there was no significant difference in the LBD abundance at the subgenome level. Since there is no standard nomenclature, the predicted TaLBDs were then named based on their chromosomal distribution and physical location.

**Table 1:** Comparison of the abundance of LBD genes in different plant species.

**Species**	**Class I**	**Class II**	**Total**
Wheat	73	17	90
Arabidopsis	36	6	42
Rice	29	6	35
Barley	19	5	24
Brachypodium	24	4	28
Maize	37	7	44
Pepper	36	9	45
Tomato	40	6	46

*LBD, lateral organ boundaries domain*.

**Table 2:** Basic information of the LBD genes identified in wheat.

**Gene name**	**EnsemblPlants ID**	**Gene length (bp)**	**ORF length (bp)**	**Deduced protein**				**Subcellular location**
				**Size (aa)**	**MW (KDa)**	**pI**	**GRAVY**	
Ta-1A-LBD1	TraesCS1A02G062200	647	621	206	21.2059	6.7	0.037378	Nuclear
Ta-1A-LBD2	TraesCS1A02G111700	1888	714	237	24.7437	8.74	−0.11772	Nuclear
Ta-1A-LBD3	TraesCS1A02G222300	808	570	189	20.4461	6.76	−0.18307	Nuclear
Ta-1A-LBD4	TraesCS1A02G260000	3986	795	264	27.1022	7.78	−0.02046	Nuclear
Ta-1B-LBD5	TraesCS1B02G080600	936	648	215	22.0948	6.61	0.012093	Nuclear
Ta-1B-LBD6	TraesCS1B02G131900	1847	711	236	24.6616	8.73	−0.13136	Nuclear
Ta-1B-LBD7	TraesCS1B02G235700	1028	570	189	20.4461	7.07	−0.18519	Nuclear
Ta-1B-LBD8	TraesCS1B02G270400	4076	792	263	27.0902	7.78	−0.03384	Nuclear
Ta-1D-LBD9	TraesCS1D02G110300	744	636	211	22.6064	8.38	−0.50237	Nuclear
Ta-1D-LBD10	TraesCS1D02G113100	1930	708	235	24.6746	8.49	−0.18596	Nuclear
Ta-1D-LBD11	TraesCS1D02G224000	996	570	189	20.4742	7.07	−0.18836	Nuclear
Ta-1D-LBD12	TraesCS1D02G259900	4097	792	263	27.0611	7.78	−0.03726	Nuclear
Ta-2A-LBD13	TraesCS2A02G194500	1442	705	234	24.2473	8.41	0.004274	Nuclear
Ta-2A-LBD14	TraesCS2A02G271300	1415	924	307	33.541	5.97	−0.64691	Nuclear
Ta-2B-LBD15	TraesCS2B02G212400	1205	714	237	24.4175	8.41	0.002954	Nuclear
Ta-2B-LBD16	TraesCS2B02G289800	1303	909	302	32.9763	6.29	−0.63576	Nuclear
Ta-2D-LBD17	TraesCS2D02G008400	1174	648	215	22.0328	6.61	0.058139	Nuclear
Ta-2D-LBD18	TraesCS2D02G193400	1336	732	243	25.7559	12.11	−0.61276	Nuclear
Ta-2D-LBD19	TraesCS2D02G270100	1619	912	303	33.0934	5.78	−0.62277	Nuclear
Ta-3A-LBD20	TraesCS3A02G093200	1898	774	257	26.492	7.72	0.02179	Nuclear
Ta-3A-LBD21	TraesCS3A02G170500	1200	723	240	25.0127	9.88	−0.54125	Nuclear
Ta-3A-LBD22	TraesCS3A02G205500	764	765	254	27.5746	7.09	−0.57126	Nuclear
Ta-3A-LBD23	TraesCS3A02G295100	1007	696	231	24.6465	7.07	−0.3316	Nuclear
Ta-3A-LBD24	TraesCS3A02G346300	1516	753	250	26.3556	6.51	−0.0492	Chloroplast
Ta-3A-LBD25	TraesCS3A02G402300	2718	780	259	26.5147	8.12	−0.02162	Nuclear
Ta-3A-LBD26	TraesCS3A02G492000	1346	1146	381	41.3431	4.57	−0.61076	Nuclear
Ta-3B-LBD27	TraesCS3B02G106900	1375	888	295	30.8182	6.42	−0.24644	Chloroplast
Ta-3B-LBD28	TraesCS3B02G108500	2073	774	257	26.49	7.72	0.046303	Nuclear
Ta-3B-LBD29	TraesCS3B02G196100	1152	747	248	25.7215	9.88	−0.56532	Nuclear
Ta-3B-LBD30	TraesCS3B02G378100	1559	879	292	30.6535	6.34	0.024657	Chloroplast
Ta-3B-LBD31	TraesCS3B02G435700	2926	780	259	26.5588	8.12	−0.00888	Nuclear
Ta-3B-LBD32	TraesCS3B02G553000	1455	1155	384	41.5733	4.93	−0.62708	Nuclear
Ta-3D-LBD33	TraesCS3D02G091700	1349	894	297	30.9474	7.07	−0.22054	Nuclear
Ta-3D-LBD34	TraesCS3D02G093500	2246	774	257	26.46	7.72	0.04786	Chloroplast
Ta-3D-LBD35	TraesCS3D02G308000	1127	741	246	26.4426	8	−0.32195	Nuclear
Ta-3D-LBD36	TraesCS3D02G340000	1516	747	248	26.0512	6.23	−0.02339	Chloroplast
Ta-3D-LBD37	TraesCS3D02G397200	3190	780	259	26.5377	8.12	−0.02317	Nuclear
Ta-3D-LBD38	TraesCS3D02G498100	1359	1155	384	41.8898	4.74	−0.61953	Nuclear
Ta-4A-LBD39	TraesCS4A02G067300	2149	801	266	27.3337	7.85	−0.22481	Nuclear
Ta-4A-LBD40	TraesCS4A02G107300	963	672	223	23.9169	6.88	−0.21794	Nuclear
Ta-4A-LBD41	TraesCS4A02G235100	1920	867	288	31.3771	7.36	−0.58854	Nuclear
Ta-4A-LBD42	TraesCS4A02G236200	2184	702	233	24.0108	7.56	−0.0588	Extracellular
Ta-4A-LBD43	TraesCS4A02G297500	3326	522	173	18.4588	8.14	−0.1659	Nuclear
Ta-4A-LBD44	TraesCS4A02G312500	993	660	219	24.1458	5.96	−0.43425	Nuclear
Ta-4A-LBD45	TraesCS4A02G415300	923	558	185	19.6217	6.98	−0.27838	Nuclear
Ta-4A-LBD46	TraesCS4A02G415400	1296	870	289	30.2694	6.74	−0.25813	Nuclear
Ta-4B-LBD47	TraesCS4B02G001200	1110	720	239	26.3001	5.97	−0.42134	Nuclear
Ta-4B-LBD48	TraesCS4B02G016200	2197	528	175	18.658	8.38	−0.196	Nuclear
Ta-4B-LBD49	TraesCS4B02G078800	1904	693	230	23.6684	7.57	−0.04957	Extracellular
Ta-4B-LBD50	TraesCS4B02G079900	4020	855	284	30.8945	7.34	−0.58099	Nuclear
Ta-4B-LBD51	TraesCS4B02G197100	1167	672	223	24.021	5.64	−0.19776	Nuclear
Ta-4B-LBD52	TraesCS4B02G224600	2045	789	262	27.1386	7.86	−0.1813	Nuclear
Ta-4B-LBD53	TraesCS4B02G316100	741	564	187	19.7849	6.7	−0.21177	Nuclear
Ta-4B-LBD54	TraesCS4B02G316200	1319	867	288	30.3994	7.29	−0.38854	Nuclear
Ta-4B-LBD55	TraesCS4B02G346500	578	507	168	18.3305	5.52	−0.42738	Nuclear
Ta-4B-LBD56	TraesCS4B02G361100	1179	882	293	30.5339	7.77	−0.19898	Chloroplast
Ta-4B-LBD57	TraesCS4B02G361200	1155	834	277	28.6916	7.79	−0.23357	Chloroplast
Ta-4D-LBD58	TraesCS4D02G001700	1042	654	217	23.8554	6.27	−0.44608	Nuclear
Ta-4D-LBD59	TraesCS4D02G014600	2210	885	294	31.8152	8.94	−0.23027	Nuclear
Ta-4D-LBD60	TraesCS4D02G077600	1454	693	230	23.7696	7.56	−0.03783	Extracellular
Ta-4D-LBD61	TraesCS4D02G078800	3868	855	284	30.8425	7.36	−0.55669	Nuclear
Ta-4D-LBD62	TraesCS4D02G197400	1112	672	223	23.9208	5.64	−0.21525	Nuclear
Ta-4D-LBD63	TraesCS4D02G225200	2152	783	260	26.9293	7.85	−0.20692	Nuclear
Ta-4D-LBD64	TraesCS4D02G312700	879	564	187	19.7428	6.7	−0.2246	Nuclear
Ta-4D-LBD65	TraesCS4D02G312800	1393	861	286	30.3505	7.21	−0.35699	Nuclear
Ta-4D-LBD66	TraesCS4D02G341500	621	522	173	19.1583	5.51	−0.49422	Nuclear
Ta-4D-LBD67	TraesCS4D02G354100	1203	897	298	31.1127	6.92	−0.17282	Chloroplast
Ta-4D-LBD68	TraesCS4D02G354200	1107	828	275	28.8449	7.77	−0.29891	Chloroplast
Ta-5A-LBD69	TraesCS5A02G152200	1695	534	177	19.7023	7.37	−0.29831	Nuclear
Ta-5A-LBD70	TraesCS5A02G191900	1574	1092	363	38.5621	5.72	−0.38843	Nuclear
Ta-5A-LBD71	TraesCS5A02G284000	652	567	188	20.1495	5.3	−0.42447	Nuclear
Ta-5A-LBD72	TraesCS5A02G515300	846	540	179	19.719	4.89	−0.42123	Nuclear
Ta-5A-LBD73	TraesCS5A02G529300	1168	870	289	30.2026	7.77	−0.23114	Chloroplast
Ta-5A-LBD74	TraesCS5A02G529400	806	807	268	27.9669	7.09	−0.23769	Chloroplast
Ta-5B-LBD75	TraesCS5B02G150800	1044	534	177	19.7163	7.37	−0.29774	Nuclear
Ta-5B-LBD76	TraesCS5B02G191200	1415	1140	379	40.1759	5.3	−0.39077	Nuclear
Ta-5B-LBD77	TraesCS5B02G282700	658	573	190	20.7041	4.94	−0.50684	Nuclear
Ta-5D-LBD78	TraesCS5D02G157400	1151	534	177	19.7183	7.37	−0.32542	Nuclear
Ta-5D-LBD79	TraesCS5D02G199000	1593	1155	384	40.6975	5.44	−0.36406	Nuclear
Ta-5D-LBD80	TraesCS5D02G291300	655	570	189	20.2766	5.28	−0.40106	Nuclear
Ta-6A-LBD81	TraesCS6A02G053700	1078	714	237	26.0791	6.49	−0.31814	Nuclear
Ta-6A-LBD82	TraesCS6A02G398400	1043	621	206	21.2541	7.99	0.151942	Nuclear
Ta-6B-LBD83	TraesCS6B02G072200	978	714	237	26.105	6.42	−0.36245	Nuclear
Ta-6B-LBD84	TraesCS6B02G438700	1033	636	211	21.7136	8	0.120379	Nuclear
Ta-6D-LBD85	TraesCS6D02G382600	1109	630	209	21.4853	7.99	0.190909	Nuclear
Ta-7A-LBD86	TraesCS7A02G066100	350	351	116	12.542	4.73	−0.17759	Nuclear
Ta-7A-LBD87	TraesCS7A02G228900	1559	690	229	24.6203	6.65	−0.38079	Nuclear
Ta-7B-LBD88	TraesCS7B02G195100	1394	690	229	24.5704	6.49	−0.27773	Nuclear
Ta-7D-LBD89	TraesCS7D02G229900	1392	690	229	24.5012	6.66	−0.35808	Nuclear
Ta-U-LBD90	TraesCSU02G132900	1125	708	235	26.0411	6.73	0.102128	Nuclear

*LBD, lateral organ boundaries domain; MW, molecular weight*.

As reported in rice and Arabidopsis, the LBD gene family could be divided into two major groups, namely, class I and class II, according to the conserved domain organization (Yang et al., 2006; Matsumura et al., 2009). We further investigated the conserved motif in these TaLBDs. Results showed that all the putative wheat LBDs possessed the conserved cysteine-rich C-motif (CX2CX6CX3C) signature motif (Supplementary Figure 1). Among them, 73 TaLBDs shared the complete cysteine-rich C-motif, GAS-block, and leucine zipper-like structure, which could be categorized into class I, while the remaining 17 TaLBDs had an incomplete leucine zipper motif, belonging to class II. Furthermore, the length of putative TaLBD proteins ranged from 116 to 384 amino acids, with the putative MW ranging from 12.5 to 41.9 KDa and theoretical PI ranging from 4.57 to 12.11, respectively. Meanwhile, the subcellular localization prediction found that most of the TaLBDs (76) were localized in the nuclear region, except for 11 in chloroplast and 3 in the extracellular region (Table 2).

### Phylogenetic Relationship, Conserved Motif, and Gene Structure Analysis

**Figure 2:**
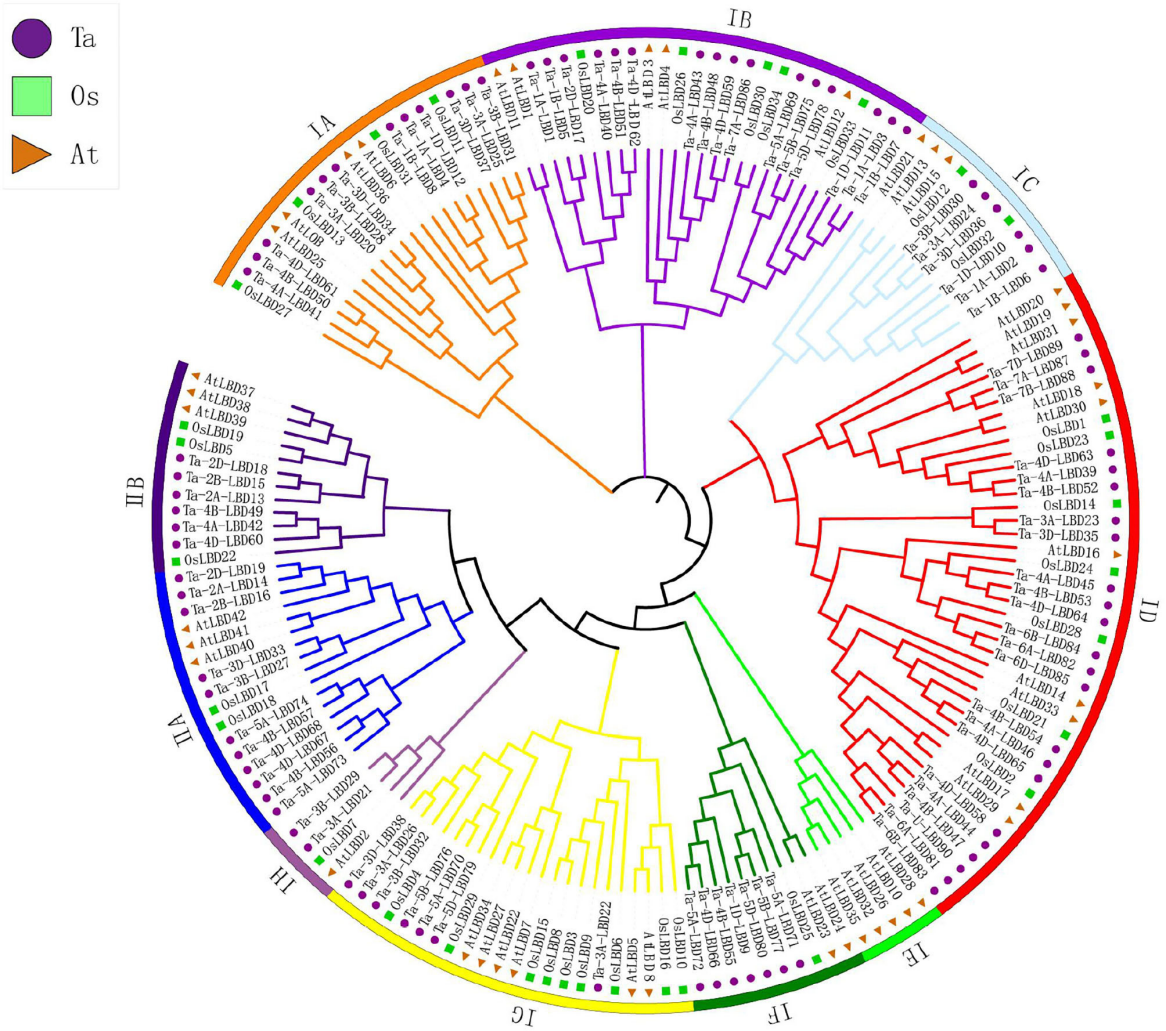
Phylogenetic analysis of LBD gene family in wheat. Tree was constructed by MEGA8.0 using the neighbor-joining method with 1,000 bootstraps. Purple, green, and orange colors represent LBD protein sequences from *Triticum aestivum* (Ta), *Oryza sativa* (Os), and *Arabidopsis thaliana* (At), respectively. The branch length represents the magnitude of genetic change. Different clade colors represent subfamilies, respectively. LBD, lateral organ boundaries domain.

**Figure 3:**
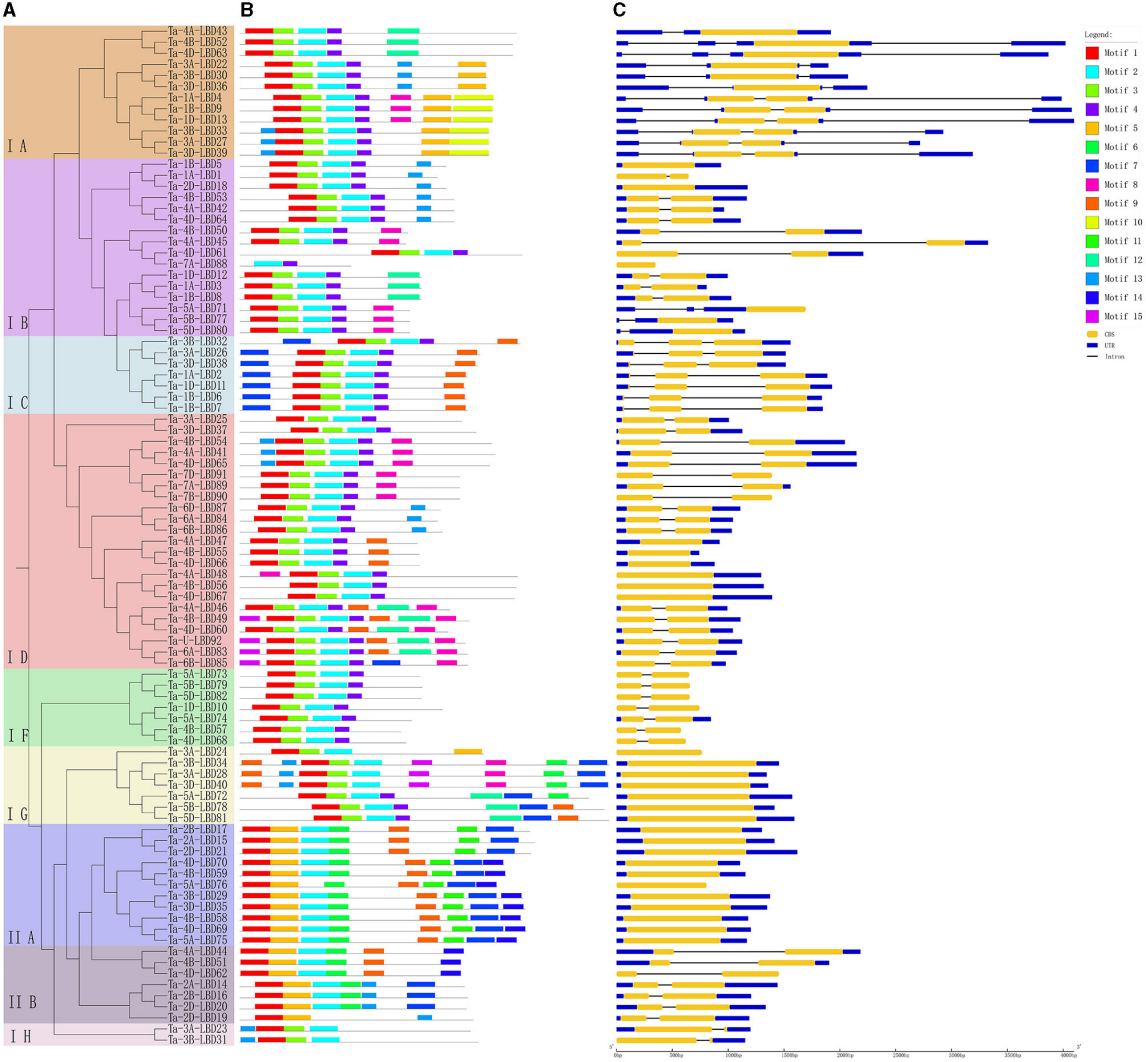
Phylogenetic relationship **(A)**, conserved motifs **(B)**, and gene structure **(C)** of LBD genes in wheat. The conserved motifs analysis of the LBDs based on their phylogenetic relationship were identified using MEME software. The different colors represent the 15 identified motifs. The yellow and blue rectangles represent the coding sequences (CDSs) and untranslated regions (UTRs), respectively, and the black lines represent introns. The lengths of the CDSs, UTRs, and introns for each LBD gene are shown proportionally. LBD, lateral organ boundaries domain.

To evaluate the evolutionary relationships of TaLBDs, phylogenetic analysis was further conducted based on multiple protein sequence alignment of all of the TaLBDs together with rice and Arabidopsis LBD proteins. Phylogenetic tree clustered these LBD genes into two major clades (class I and class II), which was consistent with the categorization depending on their domain composition as found in previous studies (Yang et al., 2006; Matsumura et al., 2009) (Figure 2). Class I could be further divided into eight groups (IA–IH), and class II was divided into two groups according to the phylogenetic relationship. It is obvious that the phylogenetic tree was monophyletic and the TaLBDs clustered together with their orthologous counterpart in rice and Arabidopsis in each subgroup, respectively (Figure 3A). Furthermore, a clear paralogous expansion by gene tandem duplication was found in the TaLBD family and each gene had two or three homoeologous copies, indicating that the allohexaploidization together with tandem duplication contributed to the expansion of TaLBDs (Qiao et al., 2015; Gombos et al., 2017). However, compared to rice and Arabidopsis, no significant paralogous expansion happened on the wheat LBD family. Interestingly, the IE subgroup only contained AtLBDs without rice and wheat orthologs, suggesting an absence of this subgroup in rice and wheat (Figure 2).

Furthermore, the deduced TaLBD proteins were submitted to the MEME web server and 15 conserved motifs were identified. Results showed that motif 1 and motif 3 were the most conserved motifs among them, which were located in the LBD domain and shared by all of the wheat LBD proteins (Figure 3B). Further analysis found that the class I and class II LBD proteins possessed a specific motif composition. All the proteins belonging to class I harbored motif 1, motif 2, and motif 3, while all the members of class II contained motif 1, motif 3, and motif 5 (Figure 3B). Interestingly, motif 5 was uniquely found in class II, which might be class II specific. It is noteworthy that the LBD proteins within the same subgroup were usually found to share a similar motifs composition. For example, motif 9 and motif 10 were shared by the three members belonging to the IE subfamily, while motif 13 was specifically shared by the nine members within the class II subfamily. These results confirmed our phylogeny-based groupings.

Gene structure can provide important clues for analyzing evolutionary features and phylogenetic relationships of a gene family (Zhu et al., 2020). Therefore, we analyzed the intron–exon structure of these TaLBDs. Results showed that most of the TaLBDs were intron-less and the number of exons ranged from 1 to 4 (Figure 3C). A total of 25 TaLBDs had no exon, 42 had one exon, and 23 genes had multiple exons. The subfamily IA had a more sophisticated structure than other subfamilies because of the various number of intron. Furthermore, the members within the same subfamily shared a similar intron–exon structure and gene size, which supported their close evolutionary relationship and the classification of subfamilies. Through comparing the LBD gene structure among wheat, Arabidopsis, and barley (Matsumura et al., 2009; Guo et al., 2016), we found that they shared similar intron–exon structures, proving that the LBD gene family was rather conserved. Based on the phylogenetic tree and gene structure, the homoeologous groups of these 90 TaLBDs were identified. A total of 38 homoeologous groups were found, of which 22 groups with each containing A, B, and D homoeologous copies, 8 groups with each containing 2 of the 3 homoeologous copies, and the remaining 8 genes had only homologous copy, suggesting that homoeologous-copy-loss event might occur in the LBD family during wheat polyploidization. The specific retention and dispersion patterns of TaLBDs in homoeologous chromosomes provided important insight into the mechanism of wheat chromosome evolution and interaction (Wang et al., 2016). Interestingly, although the chromosome location of Ta-U-LBD90 was unknown, its location could be deduced to anchor on 6D based on the homoeologous grouping.

### Cis-Elements and miRNA Targets Analysis

The cis-elements are the important regulatory factors that are involved in the transcriptional regulation of genes during plant growth and development, and stress response (Le et al., 2012). The 1.5 kb genomic sequences upstream from 5'-UTR of these 90 TaLBDs were extracted from wheat genome sequences and then used to predict cis-elements. A total of 36 cis-elements were identified belonging to different functional categories (Supplementary Tables 3 and 4). Results showed that a lot of phytohormone-responsive elements were widely found in the promoters of the TaLBDs. In detail, 78 TaLBDs contained ABA-responsive elements in their promoters, suggesting that these TaLBDs were probably induced by ABA, also 62 TaLBDs contained MeJA-responsive elements (TGACG-motif and CGTCA-motif) (Supplementary Figure 2). In addition, three gibberellin-responsive elements, namely, GARE-motif, P-box motif, and TATC-box were also found, which was consistent with the previous study that LBD genes may be involved in signaling transduction such as gibberellin pathway (Lee et al., 2009). Furthermore, light-responsive elements were also found in the promoter region of TaLBDs, for example, sp1 element and G-box element. A total of 63 out of 90 TaLBDs had the G-box element. According to the previous study, the light-responsive elements could combine with other cis-elements, such as ABA-related, to mediate signaling transduction in plant defense processes (Kumar et al., 2009), suggesting that these LBD genes might be involved in plant defense responses.

**Figure 4:**
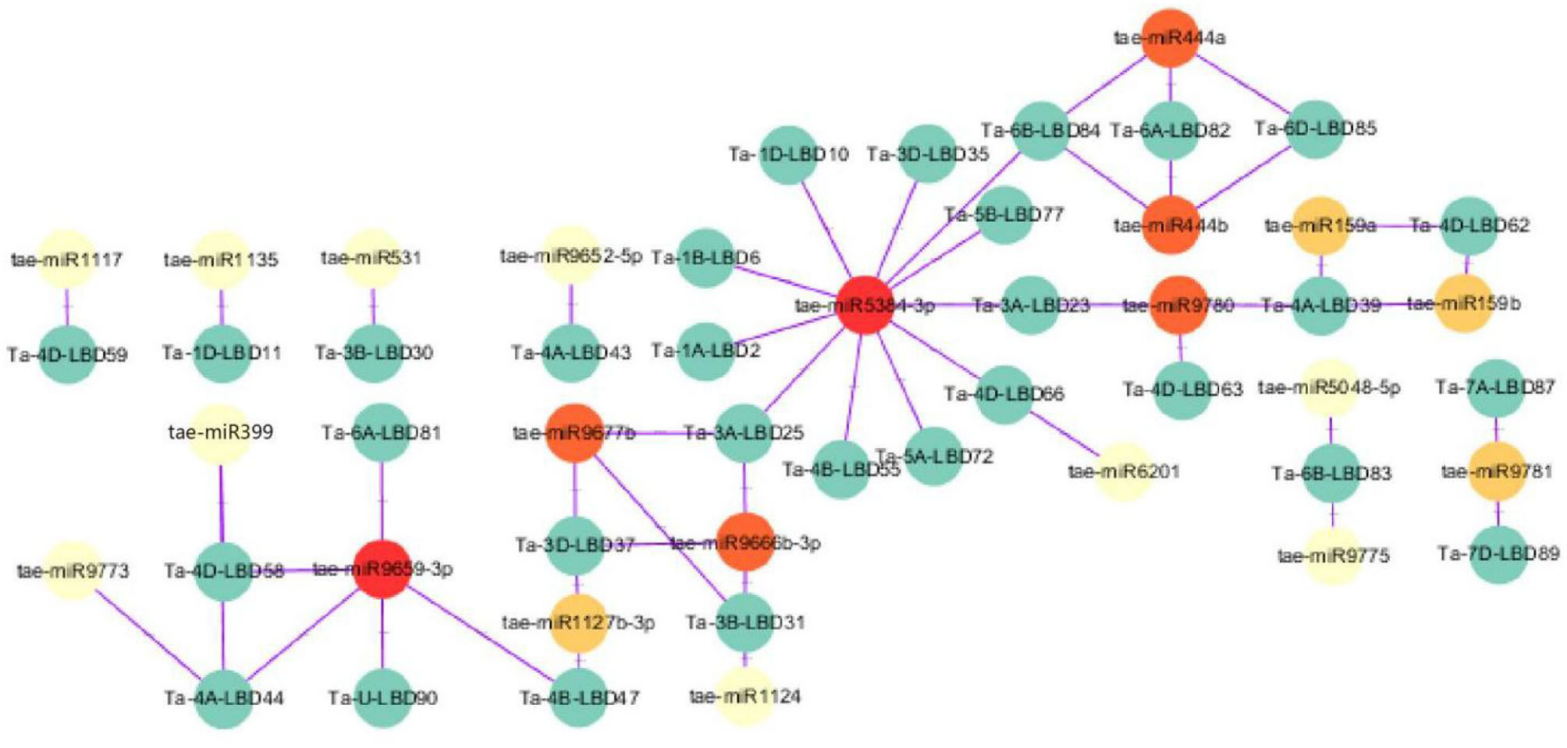
A schematic representation of the regulatory network relationships between the putative miRNAs and their targeted TaLBDs. The miRNAs that regulate one TaLBD are shown in yellow circles, miRNAs that regulate two TaLBDs are shown in orange circles, and miRNAs that regulate more than two TaLBDs are shown in red circles. The miRNAs and TaLBDs linked by the purple line indicate a putative regulatory relationship. TaLBDs, wheat lateral organ boundaries domain genes.

MicroRNAs are a class of small non-coding regulatory RNAs that are involved in controlling gene expression through guiding target mRNA cleavage or translation inhibition (Sunkar and Zhu, 2004). Recently, some miRNAs were found to regulate abiotic stress tolerance in diverse plants through targeting transcription factors (Akdogan et al., 2016; Yan et al., 2016; Zhou and Tang, 2019). To investigate the potential regulatory association between LBD transcription factor and miRNA, the putative miRNA-TaLBDs relationship was predicted using the available wheat miRNAs to search against these identified 90 TaLBDs through the psRNAtarget tool. Results showed that a total of 30 TaLBDs were predicted to be targeted by 21 miRNAs (Figure 4), of which tae-miR5384-3p could target 11 TaLBDs, tae-miR444, tae-miR9677b, and tae-miR9659-3P could target on three TaLBDs. Most of the TaLBDs were targeted by one miRNA, but Ta-4A-LBD39, Ta-3A-LBD25, Ta-3B-LBD31, and Ta-3D-LBD37 were targeted by three miRNAs. What is more, Ta-3A-LBD25, Ta-3B-LBD31, and Ta-3D-LBD37 belonged to the same homoeologous group but were targeted by three different miRNAs. The specific miRNA-LBD pairs provided the crucial information for precisely manipulating the function of these LBD genes through miRNA-based method. Although most of the TaLBDs were silenced by miRNAs through transcript cleavage, some genes were silenced by translation inhibition, such as Ta-4B-LBD55. In addition, we found that the homoeologous group genes Ta-6A-LBD82, Ta-6B-LBD84, and Ta-6D-LBD85 were regulated by tae-miR444a and tae-miR444b. According to the previous study, the miR444 family played important roles in root development, tiller formation, and stress response (Yan et al., 2014), indicating that they might be involved in wheat development and stress tolerance through miRNA regulation. The miRNAs-LBD complex identified in this study would be useful in interpreting the posttranscriptional control of gene expression during various stress-induced physiological and cellular processes in wheat and other cereal crops.

### Regulatory Network Between LBDs and Other Genes in Wheat

**Figure 5:**
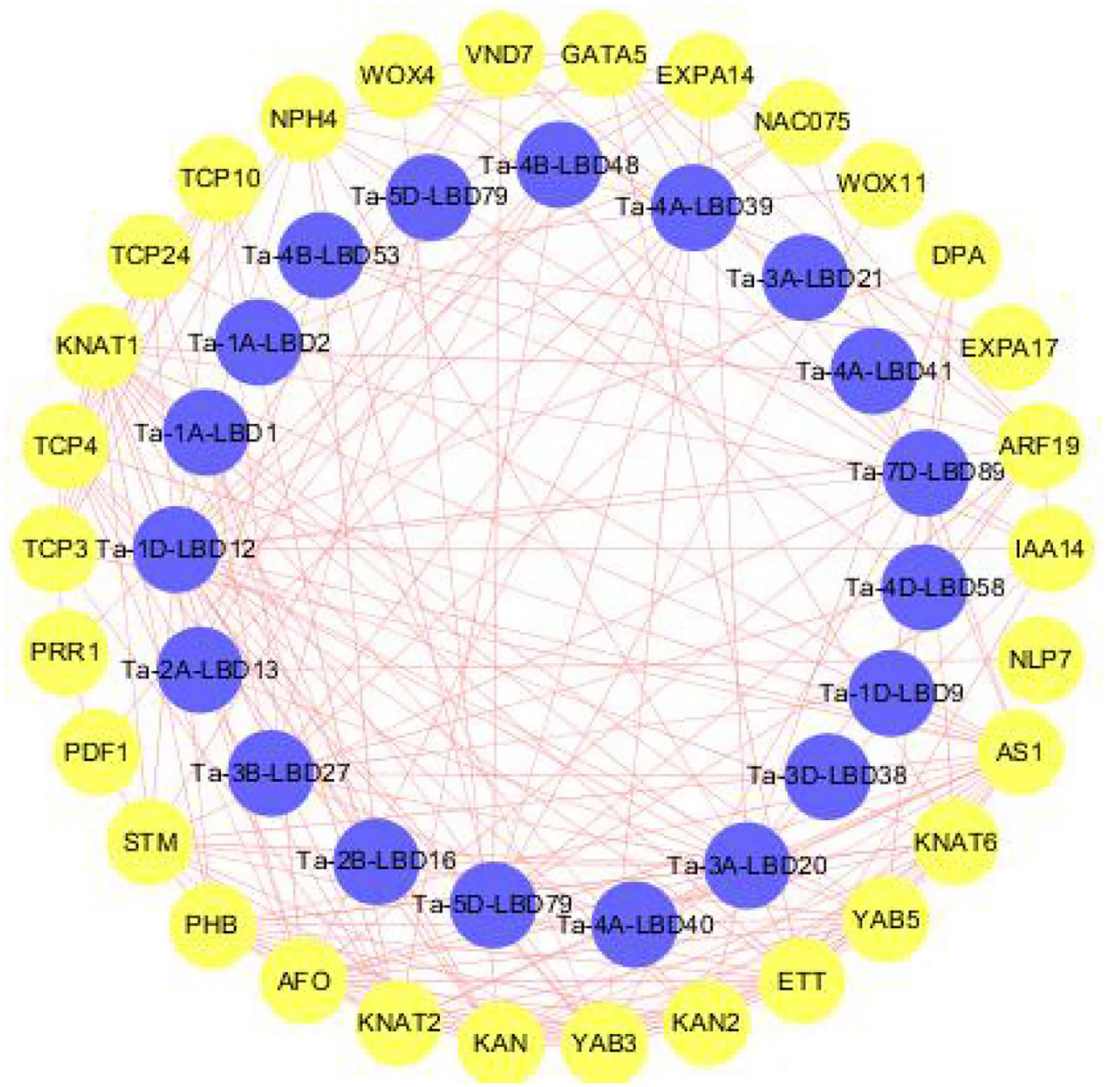
Predicted protein-protein interaction networks of TaLBD proteins with other wheat proteins using STRING tool. The blue circles represent wheat LBD proteins, and the circles on the outside represent proteins that interact with TaLBDs. The two circles connected by the red line represent the interaction between the proteins. LBD: lateral organ boundaries domain, TaLBDs, wheat lateral organ boundaries domain genes.

LBDs transcription factor superfamily plays a crucial role in regulating the complex processes of plant growth, development, and stresses response through interacting with other functional genes (Naito et al., 2007; Ma et al., 2009). To get the preliminary information about the interaction relationship between LBD and other genes in wheat, we constructed the interaction network they were involved in using the orthology-based method (Figure 5). The results revealed that these TaLBDs widely interacted with the functional genes associated with organ development and morphogenesis (KNAT, EXPA17, and PRR1) and also interacted with other transcription factors to form signal transduction cascades (ARF19, NAC075, and WOX). Among them, Ta-4A-LBD40 was found to interact with KAN and KAN2 genes, which were reported to be involved in the molecular mechanism of lateral axis-dependent development of lateral organs in seed plants (Bowman et al., 2002), suggesting that Ta-4A-LBD40 might act as a regulator in wheat lateral axis-dependent development. Previous studies found that LBD18/ASL20 could upregulate EXPA17 to promote lateral root formation through auxin signaling in Arabidopsis (Lee et al., 2009; Lee and Kim, 2013). Here, we found that Ta-3A-LBD21, the orthology of AtLBD18, could interact with EXPA17, indicating that it might regulate root development in wheat. In conclusion, the co-expression network analysis of LBD genes provided vital information for a better understanding of LBD transduction pathways in wheat.

### Expression Profiles of TaLBDs

**Figure 6:**
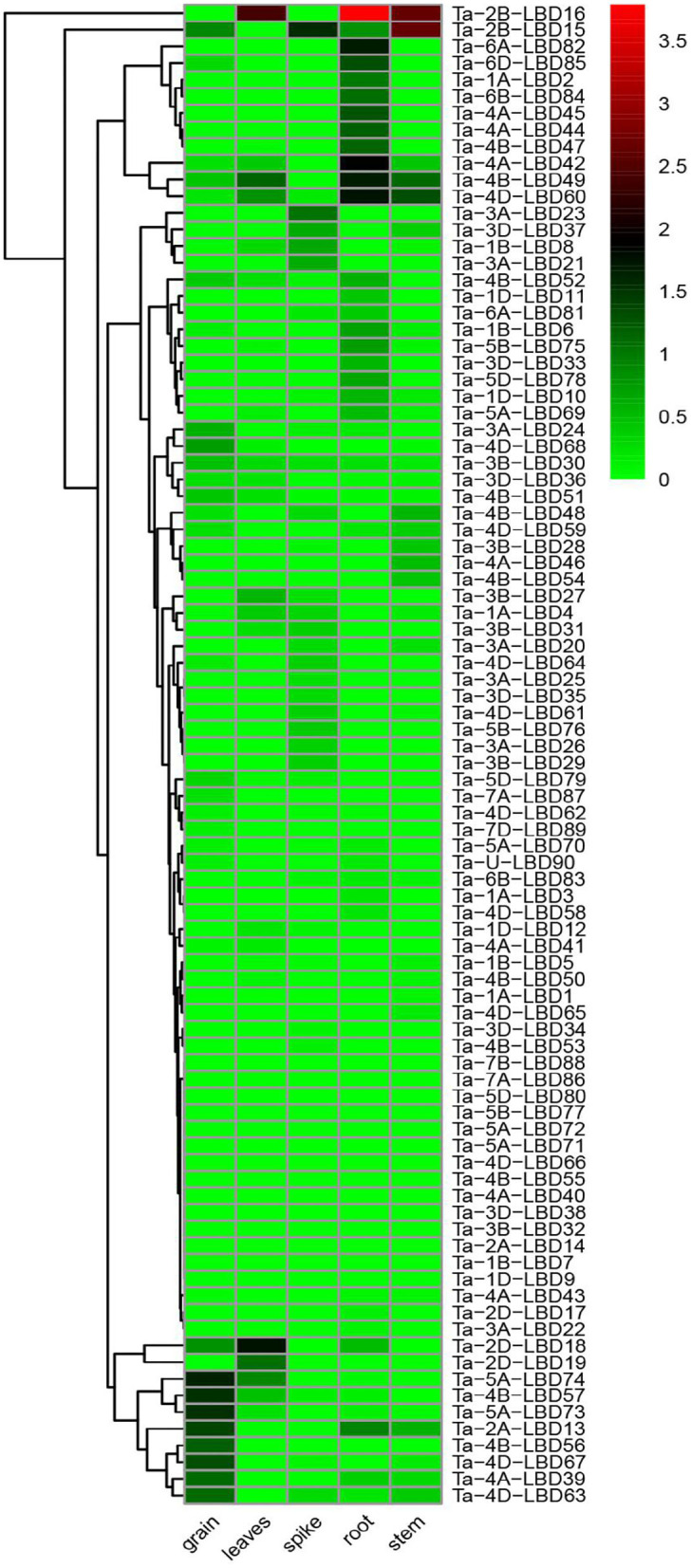
The expression profiles of 90 TaLBDs in different types of tissues, namely, grain, leaves, spike, root, and stem. The X axis represents the samples of different types of tissues. The Y axis represents the 90 different TaLBDs. The color scale represents log 2 expression values. The expression level is equal to the mean values and transforms log 2 values. TaLBDs, wheat lateral organ boundaries domain genes.

The spatiotemporal expression specificity of genes will provide helpful information to understand their function in growth and development (Tong et al., 2013). In this study, the tissue-specific expression profiles of the 90 TaLBDs in different developmental organs (grain, leaf, root, shoot, and spike) were investigated using RNA-Seq data (Figure 6). Based on the log 10-transformed (FPKM + 1) values, we found that the expression levels of TaLBDs varied significantly in different types of tissues and showed obvious tissue specificity. In detail, a total of 72 TaLBDs were detected to express in at least one of the tested tissues, while 18 showed no expression in all of these tissues. Most of the TaLBDs were much more highly expressed in the root and grain, while fewer genes showed specific expression in stem and leaves. Ta-1A-LBD2, Ta-6B-LBD84, and Ta-4B-LBD47 displayed especially high expression in root. Ta-5A-LBD73, Ta-4B-LBD56, and Ta-4D-LBD67 showed high expression in spikes, while low expression in other tissues, suggesting that they might be involved in seed development and mature. Ta-3B-LBD28, Ta-4A-LBD46, and Ta-4B-LBD54 were specially expressed in stem with relatively low levels, while Ta-2B-LBD18 displayed high expression in leaves but low expression in other tissues. Furthermore, the majority of the homoeologous genes shared similar expression patterns among different types of tissues, nevertheless, we also found that some homoeologous genes showed different expression patterns. For example, Ta-4D-LBD61 was highly expressed in spike, while its homoeologous genes Ta-4A-LBD41 and Ta-4B-LBD50 showed no expression in spike.

**Figure 7:**
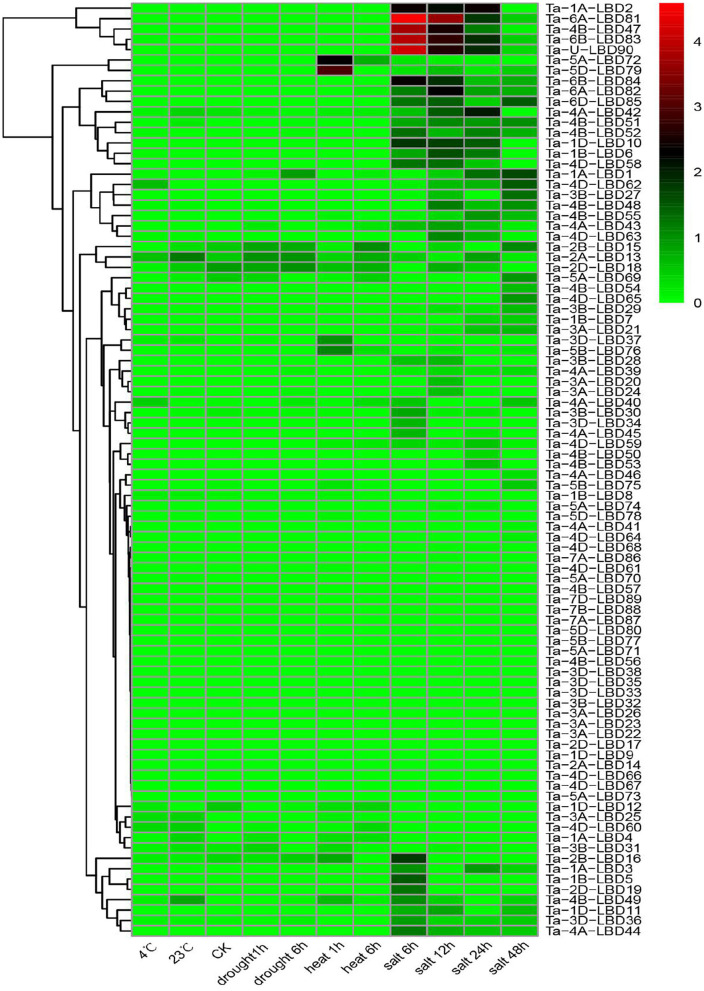
The expression profiles of 90 TaLBDs under different stresses, namely, drought, salinity, cold, and heat stresses. The X axis represents the samples of diverse stresses. The Y axis represents the 90 different TaLBDs. The color scale represents log 2 expression values. The expression level is equal to the mean values and transforms log 2 values. TaLBDs, wheat lateral organ boundaries domain genes.

Previous studies have demonstrated that the LBD genes, especially class II members, were involved in regulating stress tolerance (Kong et al., 2017; Zhang et al., 2019). To gain some insights into the expression profiles and putative functions of LBD genes in response to stresses, the expression patterns of TaLBDs in four abiotic stresses (cold, heat, drought, and salt) were investigated using RNA-seq data (Figure 7). Results revealed that most of the TaLBDs showed differential expression patterns under these abiotic stresses, particularly under salt stress. Under salt stress, 54 TaLBDs showed differential expression at different time points. Ta-6B-LBD81, Ta-4B-LBD51, and Ta-U-LBD90 displayed high expression at all of the four time points, suggesting their important role in salt stress response. Ta-2B-LBD16, Ta-2D-LBD19, and Ta-1B-LBD5 showed specifically upregulated expression at 6 h, while Ta-5A-LBD69, Ta-4B-LBD54, and Ta-4D-LBD65 expressed specifically at 24 h after salt stress. Furthermore, the expression levels of Ta-1A-LBD1 and Ta-4D-LBD62 increased continually from 6 to 24 h, while Ta-lA-LBD2, Ta-1D-LBD10, and Ta-4B-LBD47 displayed high expression at 6, 12, and 24 h and then showed downregulated expression at 48 h. These results suggested that LBD genes were widely involved in salt stress induction in wheat and the different members played differential roles in regulating the salt stress response. Under cold stress, Ta-4A-LBD40 and Ta-4D-LBD62 showed enhanced expression at 4 h while Ta-4B-LBD49 showed special expression at 23 h. In addition, Ta-2A-LBD13, Ta-2B-LBD15, and Ta-2D-LBD18 showed upregulated expression under drought stress. Ta-5A-LBD72, Ta-5D-LBD79, Ta-5B-LBD76, and Ta-3D-LBD37 showed upregulated expression at 1 h after heat stress treatment and then downregulated expression at 6 h. The spatiotemporal expression profiles of these TaLBDs provided useful information to better understand the roles of TaLBDs in the growth, development, and stress tolerance.

### Validation of the Expression of Wheat LBD Genes Using QPCR Analysis

**Figure 8:**
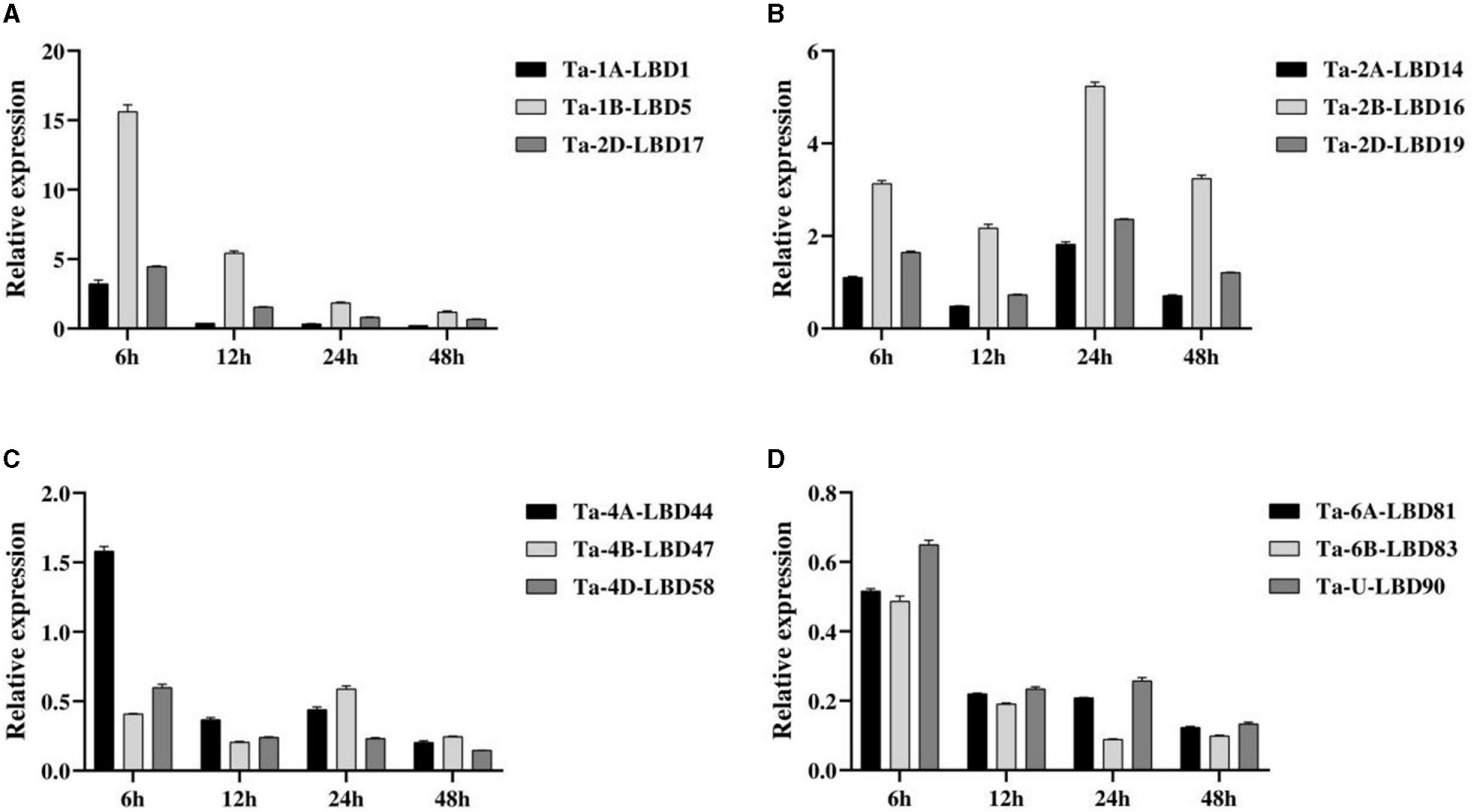
The expressions of the 12 TaLBDs belonging to four homologous groups under salt stress were investigated using QPCR method. The relative expression levels were calculated and compared. Error bars represent standard errors of three independent replicates. TaLBDs, wheat lateral organ boundaries domain genes.

Based on the expression patterns, 12 TaLBDs belonging to four homoeologous groups with significantly differential expression based on RNA-seq analysis were selected to investigate their expression levels under salt stress by QPCR analysis (Figure 8). The overall expression trend of these genes obtained by QPCR analysis was basically consistent with that of RNA-seq analysis. In detail, group A (comprising of Ta-1A-LBD1, Ta-1B-LBD5, and Ta-2D-LBD17) and group B (comprising Ta-2A-LBD14, Ta-2B-LBD16, and Ta-2D-LBD19) showed upregulated expression under salt stress, while group A showed the highest expression at 6 h and then decreased expression continually but group B showed the highest expression at 24 h after salt stress. Comparing to their A and D homoeologous genes, Ta-1B-LBD5 and Ta-2B-LBD16 showed higher expression levels at all of the four tested time points (Figures 8A,B). At the same time, the genes in group C (Ta-4A-LBD44, Ta-4B-LBD47, and Ta-4D-LBD58) and group D (Ta-6A-LBD81, Ta-6B-LBD83, and Ta-U-LBD90) showed downregulated expression under salt stress except for Ta-4A-LBD44 at 6 h (Figures 8C,D). The expression divergence among homoeologous genes was also analyzed. For group C, Ta-4A-LBD44 had the highest expression level at 6 and 12 h while its B homoeologous copy Ta-4B-LBD47 showed the highest expression level at 24 and 48 h, respectively (Figure 8C). In group D, Ta-6B-LBD83 had higher expression compared to its homoeologous genes Ta-6A-LBD81 and Ta-U-LBD90 at all of the four time points (Figure 8D). Furthermore, the expression levels of the homoeologous genes showed significant divergence, suggesting that subfunctionalization has occurred in these homoeologous genes when responding to salt stress. Further studies on the underlying effect of subfunctionalization will facilitate a better understanding of the roles of LBD genes in salt tolerance in wheat.

### Genetic Diversity of Wheat LBD Genes

To obtain information on genetic variations of TaLBDs, we further investigated their genetic diversities using the available wheat resequencing data (Zhou et al., 2020). A total of 404 (A: 183, B: 172, and D: 49) and 550 (A: 231, B: 218, and D: 101) SNPs were found in LBD genes of wheat landrace and genetically improved germplasm populations, respectively (Supplementary Table 5). The average genetic diversity (Pi value) of the landrace population was 0.889E−04, compared to that of the genetically improved germplasm population with 0.886E−04 (Supplementary Figure 3). No significant genetic diversity variation was observed between them, suggesting no severe bottleneck occurred on the LBD gene family during wheat improvement. Furthermore, at the subgenome level, the average genetic diversities of A, B, and D subgenomes in the landrace population were 1.31E−04, 1.15E−04, and 2.08E−05, respectively, while those in the genetically improved germplasm population were 1.06 E−04, 1.31E−04, and 2.87E−05 (Supplementary Table 6). The D subgenome of the genetically improved germplasm population showed higher diversity than that of the landraces, which was consistent with the previous studies based on the whole genome resequencing data (Cheng et al., 2019; Zhou et al., 2020). It demonstrated that during modern wheat breeding, genetically improved germplasm processes, introgressive hybridization with its wild D relatives had the genetic effect on the LBD gene family to enrich its diversity. At the same time, the B subgenome of the population also had higher diversity than that of landraces, which was not consistent with the general finding that landrace population owed higher diversity on B subgenome (Ormoli et al., 2015; Cheng et al., 2019; Zhou et al., 2020). These results suggested that some specific alien introgression events might occur in the TaLBDs on B subgenome. Further association of the variations with the agronomic traits will contribute to revealing the function and the evolutionary mechanism of the LBD family in wheat.

## Conclusion

This study systematically identified and characterized the LBD family in wheat. A total of 90 putative TaLBDs were obtained, which were classified into two classes based on the conserved motif signatures and phylogenetic relationship. Furthermore, the intron–exon structure and conserved motif composition of them supported the classification and the members belonging to the same subfamily shared similar gene structures. The interaction network and miRNA-TaLBDs pairs provided useful clues for revealing the LBD-mediated regulation pathway. The tissue-specific or stress-responsive TaLBDs were identified based on RNA-seq data, of which 12 were validated by QPCR analysis, proving subfunctionalization of homoeologous genes has occurred in wheat. Finally, the genetic diversity of TaLBDs showed obvious asymmetry at the subgenome level. This study not only provides the candidates for further functional analysis of the TaLBDs but also contributes to a better understanding of the regulatory mechanism of LBD genes in regulating growth, development, and stress tolerance in wheat.

## Data Availability Statement

The original contributions presented in the study are included in the article/Supplementary Material, further inquiries can be directed to the corresponding author/s.

## Author Contributions

XN and WZ conceived and designed the study. ZW and RZ performed the analysis and also drafted the manuscript. YC and PL contributed to plant material collection and QPCR analysis. WS revised the manuscript. All authors have read and approved the final manuscript.

## Conflict of Interest

The authors declare that the research was conducted in the absence of any commercial or financial relationships that could be construed as a potential conflict of interest.

## Publisher's Note

All claims expressed in this article are solely those of the authors and do not necessarily represent those of their affiliated organizations, or those of the publisher, the editors and the reviewers. Any product that may be evaluated in this article, or claim that may be made by its manufacturer, is not guaranteed or endorsed by the publisher.
