# Supplementary material for: Genome-Wide Identification, Evolution, and Expression Analysis of LBD Transcription Factor Family in Bread Wheat (Triticum aestivum L.)
Source: Front Plant Sci. 2021 Sep 3;12:721253. doi: 10.3389/fpls.2021.721253 (PMC8446603; doi:10.3389/fpls.2021.721253)
Supplement: Supplementary file 2 [file Data_Sheet_2.docx]

**Supplementary Figures**


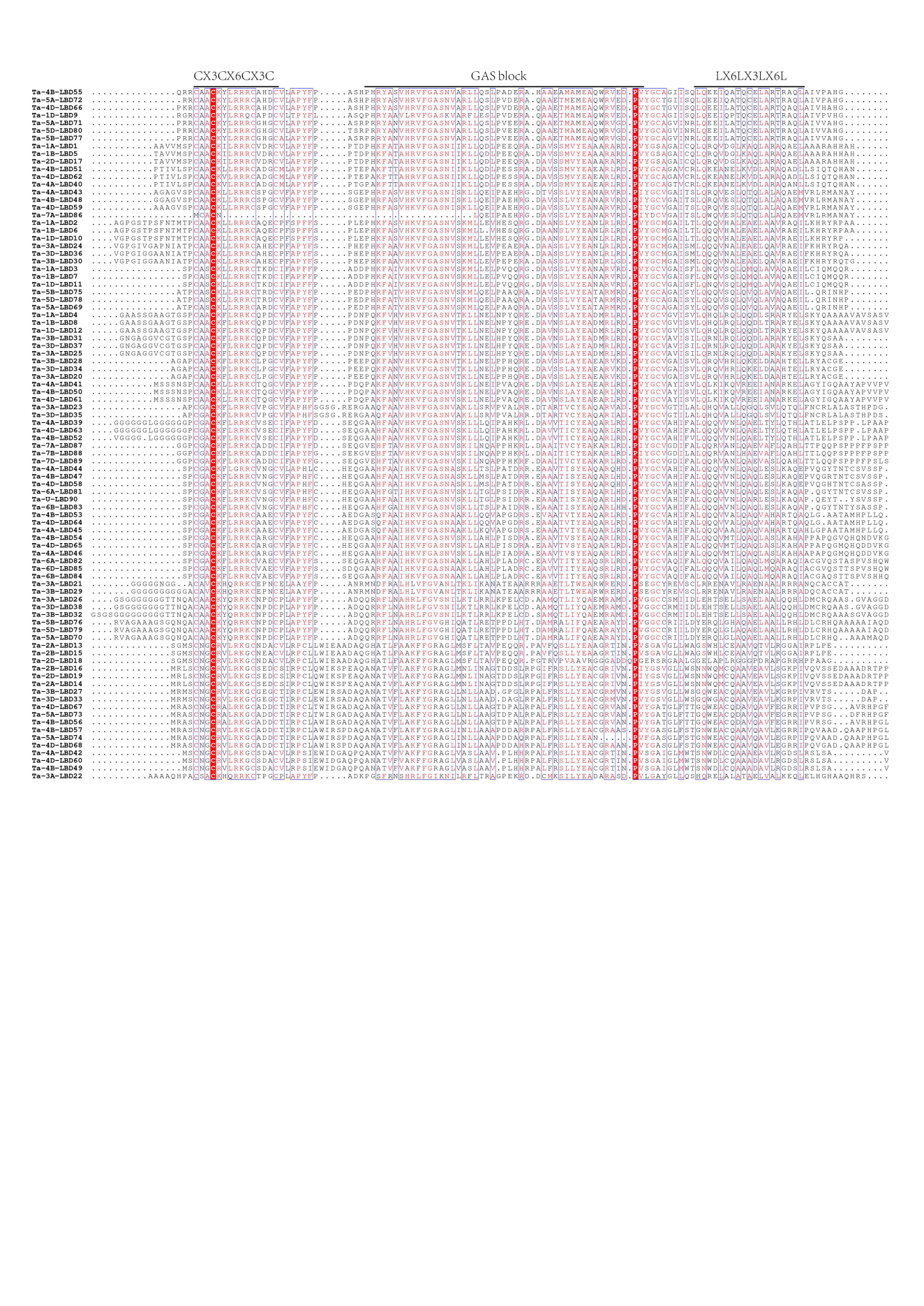
**Figure S1.** Protein sequence alignment of TaLBD genes by DNAMAN. The highlighted blue dots showed the conserved signature motif.


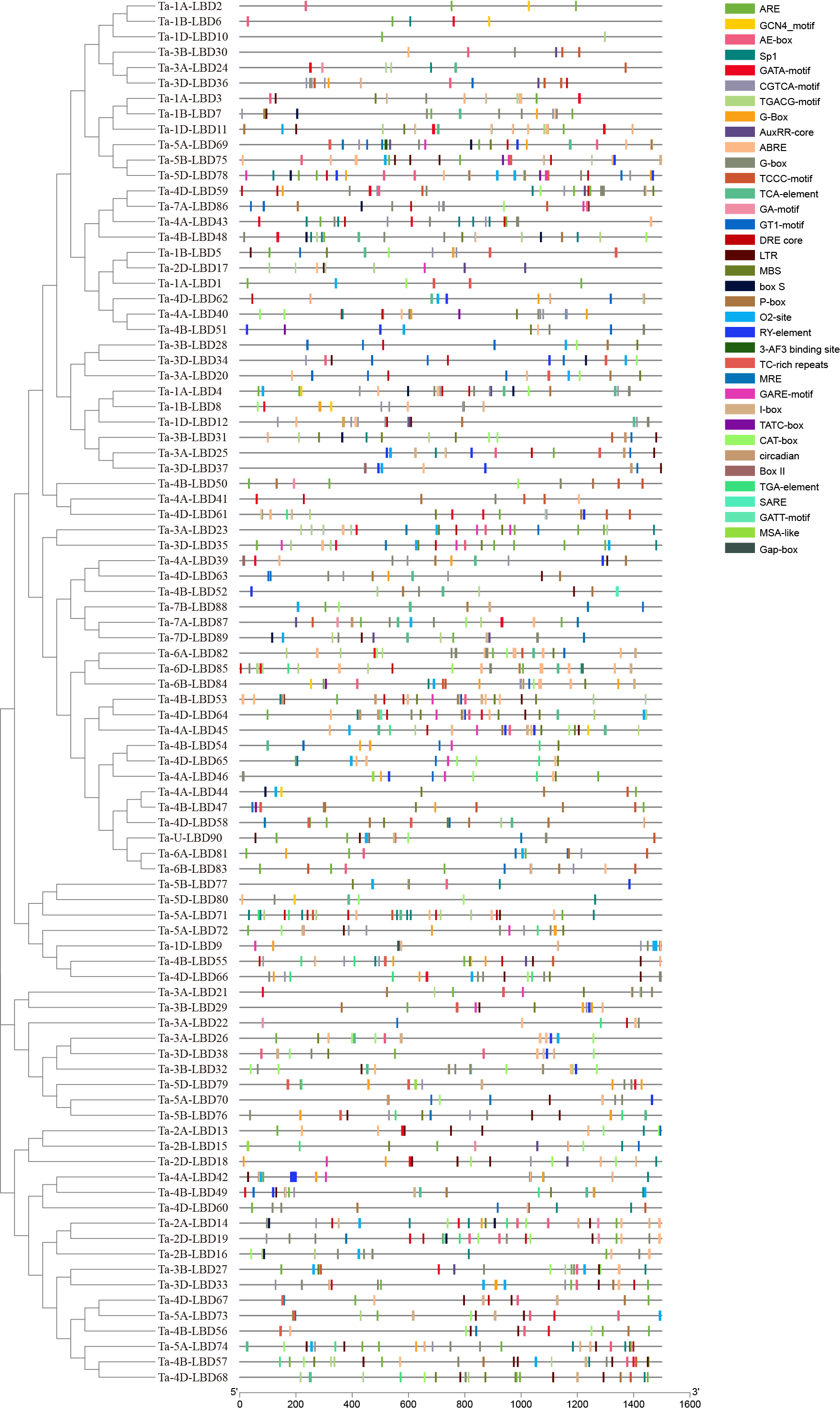


**Figure S2.** The distribution of cis-elements in 90 TaLBDs promoter regions.


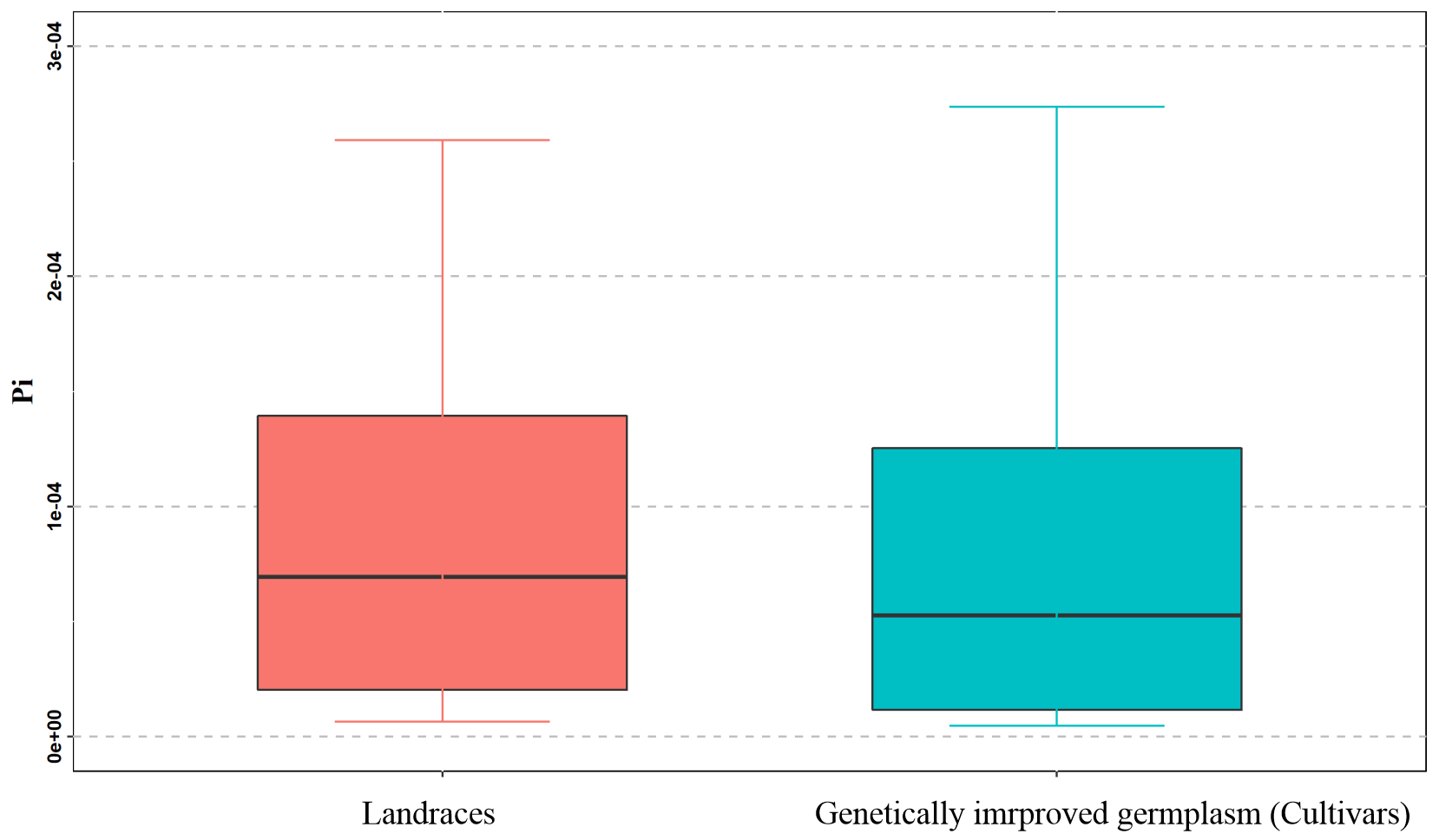


**Figure S3**. The Pi values of 90 TaLBDs in wheat landrace and genetically imrproved germplasm populations. The X axis represents the two wheat populations. The Y axis represents the Pi values. The SNP genetic variation information is obtained from the public available resequencing data provided by Zhou et al. (2020) and the accession number information is available at https://www.nature.com/articles/s41588-020-00722-w.
